# Synthesis of nonracemic hydroxyglutamic acids

**DOI:** 10.3762/bjoc.15.22

**Published:** 2019-01-25

**Authors:** Dorota G Piotrowska, Iwona E Głowacka, Andrzej E Wróblewski, Liwia Lubowiecka

**Affiliations:** 1 Bioorganic Chemistry Laboratory, Faculty of Pharmacy, Medical University of Lodz, Muszynskiego 1, 90-151 Lodz, Poland

**Keywords:** amino acids, asymmetric synthesis, chiral catalysis, chiral pool, glutamate analogues

## Abstract

Glutamic acid is involved in several cellular processes though its role as the neurotransmitter is best recognized. For detailed studies of interactions with receptors a number of structural analogues of glutamic acid are required to map their active sides. This review article summarizes syntheses of nonracemic hydroxyglutamic acid analogues equipped with functional groups capable for the formation of additional hydrogen bonds, both as donors and acceptors. The majority of synthetic strategies starts from natural products and relies on application of chirons having the required configuration at the carbon atom bonded to nitrogen (e.g., serine, glutamic and pyroglutamic acids, proline and 4-hydroxyproline). Since various hydroxyglutamic acids were identified as components of complex natural products, syntheses of orthogonally protected derivatives of hydroxyglutamic acids are also covered.

## Introduction

**Figure 1:**
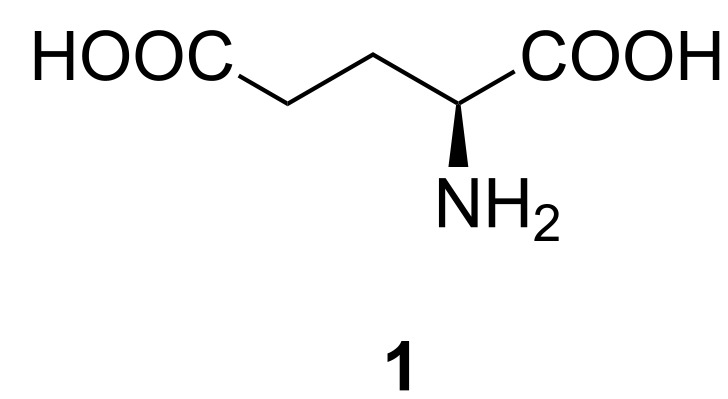
Structure of L-glutamic acid.

L-Glutamic acid (**1**, Figure 1) plays an important role in the biosynthesis of purine and pyrimidine nucleobases [1]. It also takes part in metabolic transformation to L-glutamine by L-glutamate synthetase (GS) which is crucial for cell maintenance. In neoplastic cells synthesis of L-glutamine is interfered as a result of reduced activity of GS [2]. γ-Glutamyl transpeptidase (GGT) which catalyses transfer of the γ-glutamyl group from glutathione is another enzyme relevant in cancer. High activities of GGT are observed during neoplastic transformation [3].

Several derivatives of L-glutamic acid functioning as anticancer agents have been reported [4]. But primarily L-glutamic acid is known as the major excitatory neurotransmitter in central nervous system which acts by binding to glutamate receptors [5–7]. However, these interactions are linked to several neurodegenerative diseases (Alzheimer [8], Huntington [9], Parkinson [10]) as well as to stroke [11] and epilepsy [12].

Two main classes of receptors, each of them containing three subclasses which are further divided into subtypes have been established for glutamic acid. To understand the physiological role of each receptor, recognition of their specific ligands is necessary. This, in turn, may pave a way for development of drug candidates for future therapeutic applications. These goals can be achieved by synthesis of glutamic acid analogues modifying the structure of **1** through installation of additional substituents, tuning the conformational flexibility of analogues and introducing groups capable of hydrogen bonding. Crystallographic data obtained for glutamate receptors [13–15] showed complex set of atoms interacting electrostatically and through hydrogen bonds and the conclusions from these studies should facilitate the development of new ligands.

**Figure 2:**
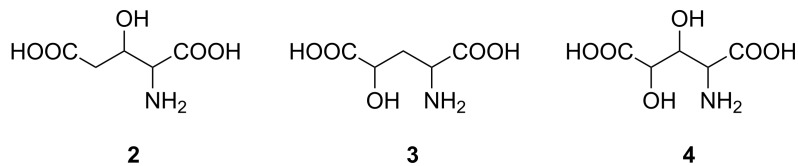
3-Hydroxy- (**2**), 4-hydroxy- (**3**) and 3,4-dihydroxyglutamic acids (**4**).

In terms of mapping of glutamate receptors hydroxyglutamic acids **2**–**4** (Figure 2) should be of great interest since an additional hydroxy group is capable of acting as a hydrogen bond donor as well as a hydrogen bond acceptor. In fact (2*S*,4*S*)-**3** showed similar potency at mGlu_1a_R and mGlu_8a_R as L-glutamic acid [16] while its affinity for AMPA and NMDA receptors was low [17]. On the other hand, (2*S*,4*R*)-**3** demonstrated significant preference for the NMDA receptor [17]. Furthermore, it was found that (2*S*,3*S*,4*S*)-**4** acts as a selective agonist of mGluR1 and as a weak antagonist of mGluR4 [18]. Excitatory amino acid transporters (EAAT) are effected by hydroxyglutamic acid in various degrees. For example, (2*S*,4*S*)-**3** appeared to be a substrate at EAAT1-3, while (2*S*,4*R*)-**3** did not interact with them [19–20]. A number of studies revealed that several giant neurons of the African giant snail appeared to be sensitive to various extents to all stereoisomers of **2** [21–23].

Hydroxyglutamic acids are widely spread in nature, especially in plants but they were also found in other species or as components of more complex molecules of interesting biological activity. Indeed, the interest in 3-hydroxyglutamic acid started many years ago by the discovery of this amino acid in hydrolysates of an antibiotic peptide S-520 [24]. It has been proved recently that it was actually the isomer (2*S*,3*R*)-**2** and it is a fragment of a cyclohexapeptide [25]. (2*R*,3*S*)-**2** and (2*R*,3*R*)-**2** were found as components of antifungal and antimicrobial hexadepsipeptides called kutznerides isolated from the actinomycete *Kutzneria* sp. 744 [26–27]. And finally, *threo*-3-hydroxyglutamic acid was identified in the cell wall of *Mycobacterium lacticum* [28].

4-Hydroxy-L-glutamic acid [(2*S*,4*S*)-**3**] was found in several plants, e.g., *Phlox decussata* [29] and other *Phlox* species [30], as well as in *Linaria vulgaris* [31]. It has also been discovered in mammalian cells as an intermediate in the degradation of hydroxyproline [32–33]. Its various amides have been identified in numerous plants [34–39] as well as components of complex molecules produced by different species [40–42]. 3,4-Dihydroxyglutamic acid (**4**) of unknown stereochemistry was identified as a constituent of seeds of *Lepidum sativum* and leaves of *Rheum rhaponticum* and later on in other species [43–44].

Natural occurrence as well as possibilities of glutamate-like biological activity modulated by additional hydrogen bonding with hydroxy groups inspired the interest in the synthesis of stereoisomers of hydroxyglutamic acids **2**–**4** (Figure 2). Since they contain two or three stereogenic centers their orthogonally protected derivatives could be considered as extremely valuable chirons in syntheses of various natural products. Their 1,2- and 1,3-aminohydroxy fragments can serve as pharmacophores of interest in medicinal chemistry. In this paper we wish to review chemical syntheses of non-racemic 3-hydroxy- (**2**), 4-hydroxy- (**3**) and 3,4-dihydroxyglutamic acid (**4**) to summarize achievements in this area. The protected forms of 3-hydroxyglutamic acid are of significant value as intermediates in the synthesis of complex peptides.

## Review

### 3-Hydroxyglutamic acid

**Figure 3:**
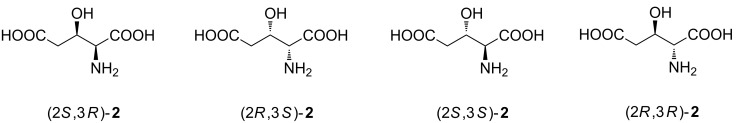
Enantiomers of 3-hydroxyglutamic acid (**2**).

The reports on the optical resolution and characterization of four enantiomers **2** (Figure 3) came from the Japanese sources [45–48]. For identification purposes (2S*,3R*)-**2** and (2S*,3S*)-**2** were resolved by chiral reversed-phase TLC [26]. Kinetic resolution of dibenzyl (2S*,3R*)-*N*-Boc-3-hydroxyglutamate was achieved in the presence of *Subtilisin Carlsberg* to give dibenzyl (2*R*,3*S*)-*N*-Boc-3-hydroxyglutamate and (2*S*,3*R*)-5-(benzyloxy)-2-[(*tert*-butoxycarbonyl)amino]-3-hydroxy-5-oxopentanoic acid [25].

The majority of asymmetric syntheses of 3-hydroxyglutamic acid employ serine or similar three-carbon chirons as starting materials. Configuration at Cα is retained in the final products and it also induces chirality at the Cβ(OH) center. The hydroxymethyl group of serine can serve as a precursor of the carboxyl fragment but when oxidized to aldehyde it may be attacked by nucleophiles to introduce the required two-carbon residue.

#### From serine-derived precursors

**Scheme 1:**
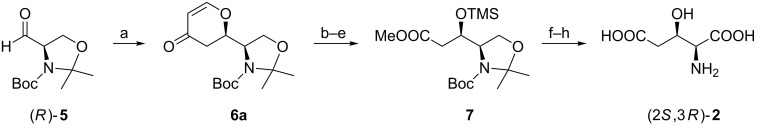
Synthesis of (2*S*,3*R*)-**2** from (*R*)-Garner's aldehyde. Reagents and conditions: a) MeOCH=CH–CH(OTMS)=CH_2_, 5% ZnCl_2_, CH_2_Cl_2_; b) NaIO_4_, RuO_2_, H_2_O; c) NaOH, H_2_O then HCl, H_2_O; d) CH_2_N_2_, ether; e) Me_3_SiNEt_2_; f) MeOH, PTSA; g) KMnO_4_, NaOH, H_2_O; h) HCl, H_2_O.

When Garner’s aldehyde (*R*)-**5** prepared from D-serine was subjected to ZnCl_2_-catalyzed cyclocondensation with Danishefsky's diene a (>9:1) mixture of diastereoisomeric pyranones **6** was formed with the *threo* isomer **6a** prevailing. Oxidative removal of two carbon atoms was followed by formate hydrolysis, formation of methyl ester and silylation to give **7** after separation from the minor diastereoisomer. After selective hydrolysis of the acetal the hydroxymethyl fragment was oxidized and all protective groups were removed to give (2*S*,3*R*)-**2** as the hydrochloride (Scheme 1). The observed stereoselectivity of the cyclocondensation step is best explained by the attack on a *re*-face of the C=O group due to chelation of Zn^2+^ to the carbonyl oxygen and amide nitrogen/oxygen atoms [49].

**Scheme 2:**
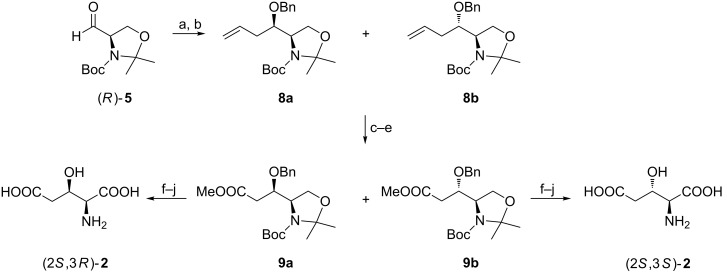
Synthesis of (2*S*,3*R*)-**2** and (2*S*,3*S*)-**2** from (*R*)-Garner’s aldehyde. Reagents and conditions: a) H_2_C=CHCH_2_MgCl, ZnCl_2_, THF; b) BnBr, NaH, THF; c) 4-methylmorpholine *N*-oxide, OsO_4_, dioxane/water; d) NaIO_4_, then NaOCl_2_, H_2_NSO_3_H; e) MeI, K_2_CO_3_, acetone; f) AcOH, H_2_O; g) NaOH, MeOH; h) PDC, DMF; i) CF_3_COOH, CH_2_Cl_2_; j) H_2_, 5% Pd/C, MeOH, H_2_O.

A better approach in terms of carbon atom economy relied on the addition of allylmagnesium chloride to the aldehyde (*R*)-**5** which after O-benzylation provided an inseparable 1:3 mixture of compounds **8a** and **8b**. A six-carbon chain was shortened by a diol formation–diol cleavage sequence followed by aldehyde oxidation and esterification to give **9a** and **9b** after chromatographic separation. They were transformed into (2*S*,3*R*)-**2** and (2*S*,3*S*)-**2** in several steps including hydroxymethyl to carboxyl oxidations (Scheme 2) [50].

**Scheme 3:**
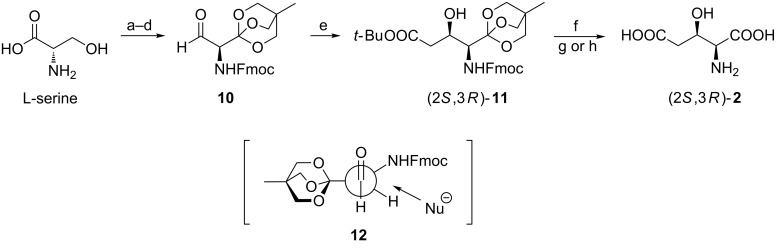
Two-carbon homologation of the protected L-serine. Reagents and conditions: a) Fmoc-succinimide, Na_2_CO_3_, dioxane, H_2_O; b) (3-hydroxymethyl)-3-methyloxetane, DCC, DMAP, CH_2_Cl_2_; c) BF_3_**·**OEt_2_, CH_2_Cl_2_; d) oxalyl chloride, DMSO, DIPEA, CH_2_Cl_2_; e) BrCH_2_COO*t*-Bu, Zn, THF, reflux; f) TMSI; g) cation exchange column, NH_4_OH; h) cation exchange column, HCl.

*N-*Fmoc protection of the amino group in L-serine together with transformation of the carboxylic function into an orthoester allow for the racemization-free oxidation to aldehyde **10**, which was immediately subjected to Reformatsky reaction to give a 92:8 mixture of (2*S*,3*R*)-**11** and (2*S*,3*S*)-**11**, respectively. The major diastereoisomer was formed by the attack on the *re*-face of the carbonyl group in accordance with the non-chelation Felkin–Ahn model **12** in which the largest substituent at Cα (orthoester) is positioned opposite to the incoming nucleophile. After removal of protective groups with iodotrimethylsilane 3-hydroxyglutamic acid (**2**) was obtained as the monoammonium salt [(2*S*,3*R*):(2*S*,3*S*), 94:6] or the hydrochloride [(2*S*,3*R*):(2*S*,3*S*), >98:2] (Scheme 3) [51].

**Scheme 4:**
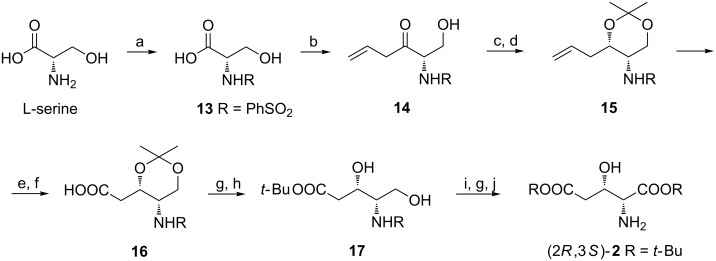
Synthesis of di-*tert*-butyl ester of (2*R*,3*S*)-**2** from L-serine. Reagents and conditions: a) PhSO_2_Cl, K_2_CO_3_, H_2_O; b) BuLi, THF; then H_2_C=CHCH_2_MgBr; c) L-selectride, THF; d) Me_2_C(OMe)_2_, PTSA, THF; e) O_3_, CH_2_Cl_2_, ether, then Ph_3_P; f) KMnO_4_, acetone, H_2_O; g) *t*-BuOH, *N*,*N*′-diisopropyl-*O*-*tert*-butylisourea, CH_2_Cl_2_; h) MeOH, HCl (gas); i) O_2_, Pt, AcOEt, H_2_O; j) electrochemical reduction.

Treatment of a trilithium salt of the N-protected acid **13** derived from L-serine with allylmagnesium bromide provided ketone **14** which was reduced to diastereoisomeric diols in a 9:1 *syn* to *anti* ratio when L-selectride was applied. They were separated as isopropylidene derivatives and the *syn* isomer **15** was subjected to ozonolysis and oxidation to give acid **16**. To complete the synthesis of di-*tert*-butyl ester of (2*R*,3*S*)-**2** compound **16** was first transformed into the ester and later deprotected to the diol **17** which was selectively oxidized, again esterified and finally the phenylsulfonyl group was removed electrochemically (Scheme 4) [52].

**Scheme 5:**
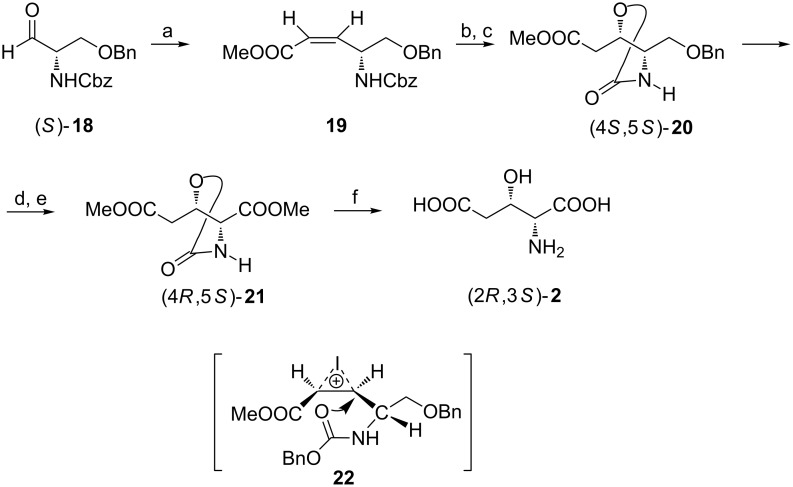
Synthesis of (2*R*,3*S*)-**2** from *O*-benzyl-L-serine. Reagents and conditions: a) (CF_3_CH_2_O)_2_P(O)CH_2_COOMe, KHMDS, 18-crown-6; b) I_2_, MeCN; c) Bu_3_SnH, AIBN, benzene, reflux; d) H_2_, 10% Pd/C, ethanol; e) CrO_3_, acetone, then CH_2_N_2_, ether; f) 3 M HCl, 80 °C.

The aldehyde (*S*)-**18** prepared from *O*-benzyl-L-serine in three standard steps [53] was elongated by a two-carbon fragment employing a Wittig reaction to give *Z*-alkene **19**. To introduce the next center of chirality of the required configuration a iodocyclocarbamation reaction was applied to give *trans*-oxazolidin-2-one (4*S*,5*S*)-**20** after reduction of the carbon–iodine bond formed in the primary products of cyclization (via iodonium ion **22**). Hydrogenolytic debenzylation preceded oxidation of the hydroxymethyl group to afford diester (4*R*,5*S*)-**21** which after hydrolysis gave (2*R*,3*S*)-**2** as the hydrochloride (Scheme 5) [53]. Starting from *O*-benzyl-D-serine (2*S*,3*R*)-**2** was obtained in a similar way.

**Scheme 6:**
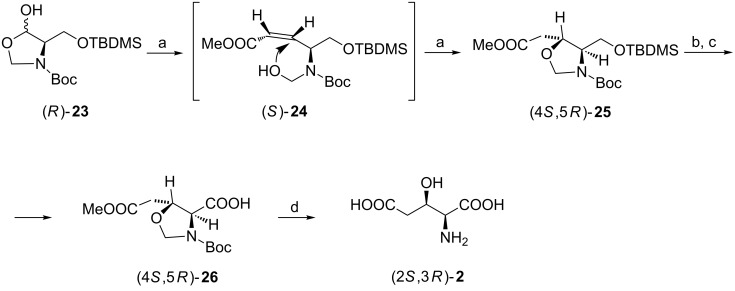
Synthesis of (2*S*,3*R*)-**2** employing a one-pot *cis*-olefination–conjugate addition sequence. Reagents and conditions: a) (CF_3_CH_2_O)_2_P(O)CH_2_COOMe, KHMDS, 18-crown-6, THF; b) PTSA, MeOH; c) NaOCl, TEMPO, KBr, NaHCO_3_, water/acetone; d) 3 M HCl, 80 °C.

Configurationally stable D-serinal derivative (*R*)-**23** (prepared from D-serine [54]) which primarily exists as hemiacetal was subjected to *cis*-olefination with Stille's reagent at −30 °C to produce (*S*)-**24** in good yield. However, when the reaction mixture was warmed to 0 °C before quenching, an intramolecular cyclization occurred under basic conditions to give the oxazolidine (4*S*,5*R*)-**25** as an almost (>20:1) pure diastereoisomer. The hydroxy group which acted as a nucleophile preferred to attack the *re*-face of the double bond for steric reasons. Selective removal of the silyl protective group allowed for the hydroxymethyl to carboxyl transformation to (4*S*,5*R*)-**26**, and hydrolysis afforded (2*S*,3*R*)-**2** as the hydrochloride (Scheme 6) [55].

#### From homochiral aziridine

**Scheme 7:**
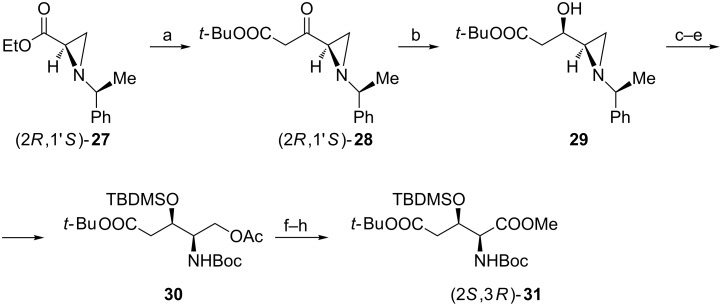
Synthesis of the orthogonally protected (2*S*,3*R*)-**2** from a chiral aziridine. Reagents and conditions: a) LiHMDS, AcO*t*-Bu, THF; b) NaBH_4_, iPrOH; c) *t*-BuMe_2_SiCl, TEA, DMAP, CH_2_Cl_2_; d) AcOH, CH_2_Cl_2_; e) H_2_, 10% Pd(OH)_2_, Boc_2_O, MeOH; f) KOH, EtOH; g) NaIO_4_, RuCl_3_, CCl_4_/MeCN/H_2_O; h) MeI, KHCO_3_, DMF.

An interesting approach to protected (2*S*,3*R*)-**2** makes use of the aziridine (2*R*,1′*S*)-**27** as a synthetic equivalent of L-serine (Scheme 7) [56]. Stereoselective reduction of ketone (2*R*,1′*S*)-**28** gave hydroxyaziridine **29** as the major (10:1) product which, after the protection of the hydroxy group, was subjected to the regioselective aziridine ring opening, catalytic removal of the chiral auxiliary with simultaneous formation of a *N*-Boc derivative **30**. The hydroxymethyl to carboxylate transformation to form the protected diester (2*S*,3*R*)-**31** required prior basic deacetylation followed by standard oxidation and esterification. Diastereoisomer (2*S*,3*S*)-**31** was also prepared employing the same methodology.

#### From *N*-Boc-D-phenylglycinal

**Scheme 8:**
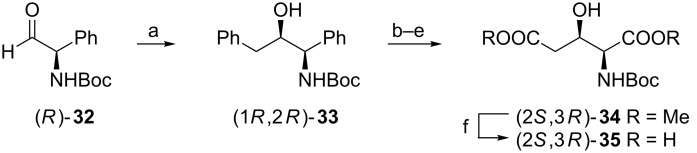
Synthesis of *N-*Boc-protected (2*S*,3*R*)-**2** from D-phenylglycine. Reagents and conditions: a) BnMgCl, ether; b) Me_2_C(OMe)_2_, PPTS, toluene; c) NaIO_4_, RuCl_3_, NaHCO_3_, CCl_4_/MeCN/H_2_O; d) CH_2_N_2_, ether; e) 80% AcOH; f) LiOH, H_2_O/THF/MeOH.

Since the phenyl group has been applied for many occasions as a precursor of the carboxylic function selection of D-phenylglycine as a starting material in the synthesis of the *N-*Boc-protected (2*S*,3*R*)-**2** makes a useful addition to the existing methodologies (Scheme 8) [57]. Thus, *N*-Boc-D-phenylglycinal (*R*)-**32** was in situ treated with benzylmagnesium chloride to give *N*-Boc-aminoalcohol (1*R*,2*R*)-**33** as a major (9:1) product easily separable chromatographically. Before oxidative degradation of both phenyl groups (1*R*,2*R*)-**33** was protected as an acetonide. Intermediary diacid was first esterified with diazomethane, then the isopropylidene acetal was hydrolyzed, and diester saponification gave *N*-Boc-protected compound (2*S*,3*R*)-**35**.

#### Via ketopinic acid functionalized 2(3*H*)-oxazolones

**Scheme 9:**
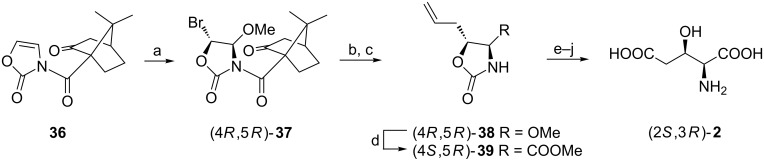
Synthesis of (2*S*,3*R*)-**2** employing ketopinic acid as chiral auxiliary. Reagents and conditions: a) Br_2_, MeC(OMe)_3_, Me_3_SiOTf, CH_2_Cl_2_; b) H_2_C=CHCH_2_SnBu_3_, *h*ν; c) Bu_2_CuLi; d) Me_3_SiCN, TiCl_4_, CH_2_Cl_2_, then MeOH, HCl; e) Boc_2_O, NaH, THF; f) Cs_2_CO_3_, MeOH; g) *t*-BuMe_2_SiCl, imidazole, DMF; h) NaIO_4_, KMnO_4_, then CH_2_N_2_, ether; i) TBAF; j) 6 M HCl.

When oxazolone **36** derived from (*R*)-(−)-ketopinic acid was reacted with bromine and trimethyl orthoacetate the enantiomerically pure bromomethoxy derivative (4*R*,5*R*)-**37** was prepared after crystallization of the reaction mixture. The precursor of the carboxymethyl group was first introduced with full retention of configuration employing a stannate chemistry to give (4*R*,5*R*)-**38** after removal of a chiral auxiliary with lithium dibutylcuprate. Next, titanium tetrachloride-catalyzed cyanation secured another carboxy group and after a few transformations an oxazolidinone (4*S*,5*R*)-**39** was obtained as a major (7:1) product readily purified chromatographically. To complete the synthesis of (2*S*,3*R*)-**2** *N-*Boc protection preceded the cleavage of the oxazolidine ring while silylation of the hydroxy group was necessary before oxidation of the C=C bond (Scheme 9) [58].

**Scheme 10:**
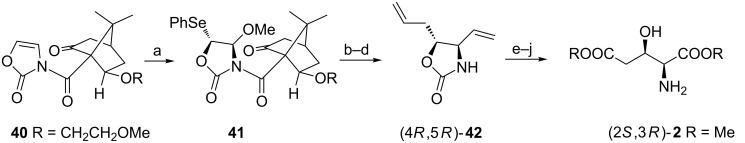
Synthesis of dimethyl ester of (2*S*,3*R*)-**2** employing (1*S*)-2-*exo*-methoxyethoxyapocamphane-1-carboxylic acid as a chiral auxiliary. Reagents and conditions: a) PhSeCl, MeOH; b) H_2_C=CHCH_2_SnBu_3_, ether, *h*ν; c) BnSLi, THF; d) (H_2_C=CH)_2_CuCNMgBr, BF_3_**·**OEt_2_, THF; e) Boc_2_O, DMAP, THF; f) Cs_2_CO_3_, MeOH; g) Me_2_C(OMe)_2_, PTSA, benzene; h) NaIO_4_, KMnO_4_, water/acetone; i) CH_2_N_2_, ether; j) MeOH, HCl (gas).

Further applications of the ketopinic acid framework as a chiral auxiliary relied on fine tuning of the steric environment around the carbonyl group. Thus, when compound **40** prepared using readily available (*S*)-(+)-ketopinic acid was reacted with phenylselenyl chloride in methanol the adduct **41** was formed with high diastereoselectivity (de 96%) and was later separated chromatographically. Further transformations into dimethyl ester of (2*S*,3*R*)-**2** involved attachment of allyl and vinyl groups to form (4*R*,5*R*)-**42** which was next oxidized to diacid and finally esterified to give dimethyl ester of (2*S*,3*R*)-**2** as the hydrochloride (Scheme 10) [59].

#### By formation of the pyrrolidine ring

**Scheme 11:**
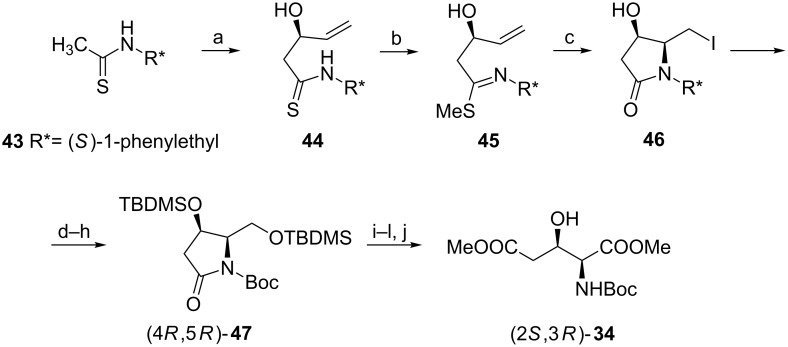
Synthesis of *N-*Boc-protected dimethyl ester of (2*S*,3*R*)-**2** from (*S*)-*N*-(1-phenylethyl)thioacetamide. Reagents and conditions: a) BuLi, THF, then H_2_C=CHCHO; b) MeI, K_2_CO_3_, acetone; c) I_2_, THF; d) EtCOO**^–^** Cs^+^, DMF; e) K_2_CO_3_, EtOH; f) *t*-BuMe_2_SiCl, imidazole, DMAP, DMF; g) Na, liquid NH_3_; h) Boc_2_O, NaH, THF; i) KOH, MeOH/H_2_O; j) CH_2_N_2_, ether; k) TBAF, THF; l) O_2_, Pt.

Important synthetic strategies towards 3-hydroxyglutamic acids take advantage of the intermediary formation of the pyrrolidine ring. Addition of the dianion of **43** to acrolein gave a 69:31 mixture of diastereoisomers with compound **44** predominating which was easily separated on silica gel. When imine **45** was treated with iodine a stereoselective iodolactamization occurred to produce lactam **46** having the same configurations as found in (2*S*,3*R*)-**2**. To complete the synthesis of (2*S*,3*R*)-**34** first the iodomethyl group was transformed in two steps into the hydroxymethyl moiety, both hydroxy groups were silylated, the chiral auxiliary was removed and the amide nitrogen was protected as *N-*Boc to furnish (4*R*,5*R*)-**47**. Under basic conditions the pyrrolidin-2-one ring was cleaved to provide a five-carbon chain of the target molecule. The final steps included esterification, desilylation and selective oxidation of the hydroxymethyl group followed by esterification (Scheme 11) [60].

**Scheme 12:**
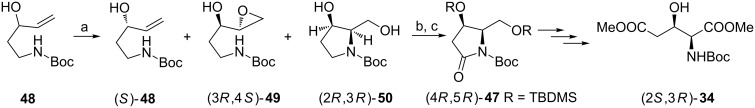
Synthesis of *N-*Boc-protected dimethyl ester of (2*S*,3*R*)-**2** via Sharpless epoxidation. Reagents and conditions: a) TBHP, D-(−)-DIPT, Ti(OiPr)_4_, MS, CH_2_Cl_2_; b) *t*-BuMe_2_SiCl, imidazole, DMAP, DMF; c) NaIO_4_, RuO_2_, AcOEt/H_2_O.

Sharpless epoxidation of the allylic alcohol **48** gave a 46:11:33 mixture of (*S*)-**48**, (3*R*,4*S*)-**49** and (2*R*,3*R*)-**50**. While (*S*)-**48** is a product of kinetic resolution, the formation of (2*R*,3*R*)-**50** results from the intramolecular opening of the oxirane ring in (3*R*,4*S*)-**49**. After chromatographic separation the hydroxy groups in (2*R*,3*R*)-**50** were protected as silyl ethers to allow oxidation at C5 to produce pyrrolidine-2-one (4*R*,5*R*)-**47** (Scheme 12) which was later transformed into (2*S*,3*R*)-**34** as already shown (Scheme 11) [61].

#### From L-malic acid

**Scheme 13:**
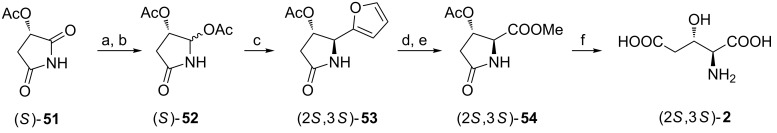
Synthesis of (2*S*,3*S*)-**2** from the imide **51**. Reagents and conditions: *a)* NaBH_4_, MeOH/CH_2_Cl_2_; b) Ac_2_O, pyridinium perchlorate; c) furan, ZnCl_2_, Me_3_SiCl, MeNO_2_; d) O_3_, MeOH; e) CH_2_N_2_, ether; f) 6 M HCl.

(*S*)-Acetoxypyrrolidin-2,5-dione (**51**), readily available from L-malic acid [62], was carefully reduced and immediately acetylated to (*S*)-**52** which was reacted with furan to produce a 67:33 mixture of readily separable (2*S*,3*S*)-**53** and (2*R*,3*S*)-**53**, respectively. Steric hindrance of the acetoxy substituent controls the formation of higher amounts of the *trans*-isomer. Ozone efficiently completed the degradation of the furan ring to the carboxyl group which was esterified with diazomethane to give methyl (2*S*,3*S*)-3-acetoxypyroglutamate (2*S*,3*S*)-**54**, a cyclized variant of 3-hydroxyglutamic acid. Treatment with a concentrated acid afforded (2*S*,3*S*)-**2** as the hydrochloride (Scheme 13) [63].

**Scheme 14:**
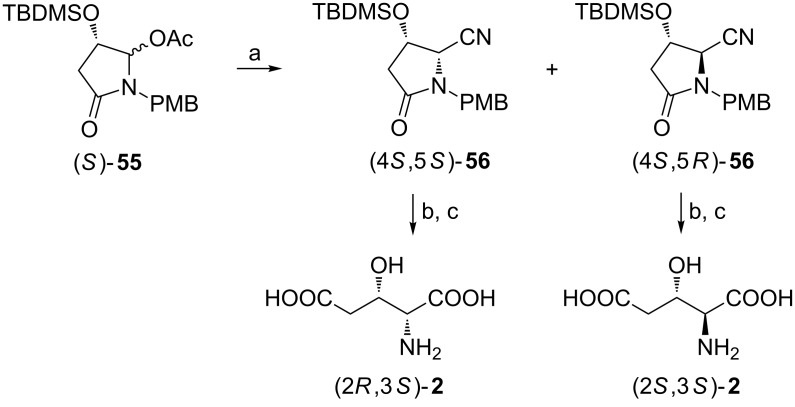
Synthesis of (2*R*,3*S*)-**2** and (2*S*,3*S*)-**2** from the acetolactam **55** (PMB = *p*-methoxybenzyl). Reagents and conditions: a) Me_3_SiCN, BF_3_**·**OEt_2_, CH_2_Cl_2_; b) Ce(NH_4_)_2_(NO_3_)_6_, MeCN; c) 6 M HCl, then Dowex 50W-X8.

The other strategy which also commences from L-malic acid [64] showed much better carbon atom economy since the acetate (*S*)-**55** was reacted with cyanide while to the acetate (*S*)-**52** three unused carbon atoms were added. The cyanides (4*S*,5*S*)-**56** and (4*S*,5*R*)-**56** formed as a 57:43 mixture in a boron trifluoride-catalyzed reaction with trimethylsilyl cyanide were separated chromatographically. Their transformation into (2*R*,3*S*)-**2** and (2*S*,3*S*)-**2**, respectively, required the removal of the *p*-methoxybenzyl group and an acidic hydrolysis (Scheme 14) [65].

#### From D-glucose

**Scheme 15:**
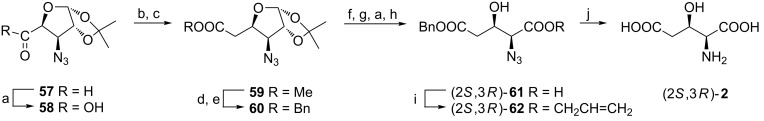
Synthesis of (2*S*,3*R*)-**2** from D-glucose. Reagents and conditions: a) NaClO_2_, 30% H_2_O_2_, NaH_2_PO_4_, MeCN; b) ClCOOEt, NEt_3_, then CH_2_N_2_, ether; c) MeOH, PhCOOAg, NEt_3_; d) LiOH, THF/H_2_O; e) ClCOOBn, NEt_3_, DMAP; f) TFA, H_2_O; g) NaIO_4_, acetone/water; h) NaHCO_3_, THF/H_2_O; i) H_2_C=CHCH_2_Br, NaHCO_3_, DMF; j) H_2_, 10% Pd/C, MeOH/HCl.

D-Glucose may be used as a chiral template for the synthesis of (2*S*,3*R*)-**2** since configurations at C3 and C4 in the hexose are retained in the target compound. The disclosed strategy relied on prior transformation of D-glucose into azidofuranoside **57** [66] and next to acid **58**. Homologation of acid **58** was accomplished by the Arndt–Eistert reaction to give the methyl ester **59** from which benzyl ester **60** was obtained for easy hydrogenolytic removal in the last step. Hydrolysis of the isopropylidene acetal was followed by periodate cleavage of the C1–C2 bond in the furanose, oxidation of the already formed aldehyde to the acid and basic hydrolysis of the formate to afford the acid (2*S*,3*R*)-**61**. Its allylation provided the ester (2*S*,3*R*)-**62**, a protected precursor of 3-hydroxyglutamate, from which (2*S*,3*R*)-**2** can be prepared by catalytic hydrogenolysis (Scheme 15) [67].

### 4-Hydroxyglutamic acids

**Figure 4:**
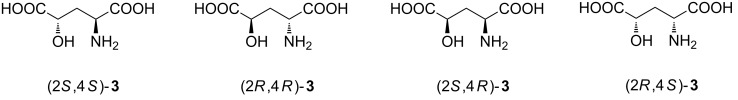
Enantiomers of 3-hydroxyglutamic acid (**3**).

All enantiomers of 4-hydroxyglutamic acid (**3**, Figure 4) were synthesized and characterized [68–69] and absolute configurations were established [68–69].

Although the majority of their preparations rely on enzymatic processes [5,68,70–73] several syntheses of non-racemic 4-hydroxyglutamic acids have been elaborated.

#### By electrophilic hydroxylation at C4

**Scheme 16:**
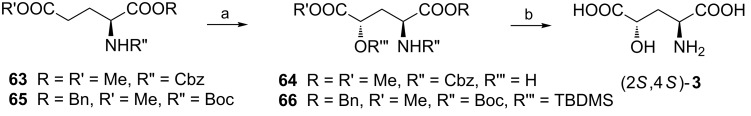
Synthesis of (4*S*)-4-hydroxy-L-glutamic acid [(2*S*,4*S*)-**3**] by electrophilic hydroxylation. Reagents and conditions: a) 3-phenyl-*N*-phenylsulfonyl oxaziridine, then LiHMDS, THF; b) 6 M HCl.

When the lithium enolate of dimethyl *N*-Cbz-L-glutamate **63** was treated with Davis oxaziridine, an inseparable 9:1 mixture of diastereoisomers was formed with (2*S*,4*S*)-**64** predominating (Scheme 16) [74]. For sodium and potassium enolates diastereoselectivity of the hydroxylation was much lower (2.6:1 and 1:1, respectively). Acid hydrolysis of **64** gave (4*S*)-4-hydroxy-L-glutamic acid [(2*S*,4*S*)-**3**] as the hydrochloride, however, its enantiomeric purity was not checked.

In connection with the total synthesis of thiopeptide antibiotic nosiheptide an orthogonally protected (4*S*)-4-hydroxy-L-glutamic acid derivative **66** (Scheme 16) was required and it was obtained as a single diastereoisomer from **65** in the same way [75–76].

#### By bromination of L-glutamic acid

**Scheme 17:**
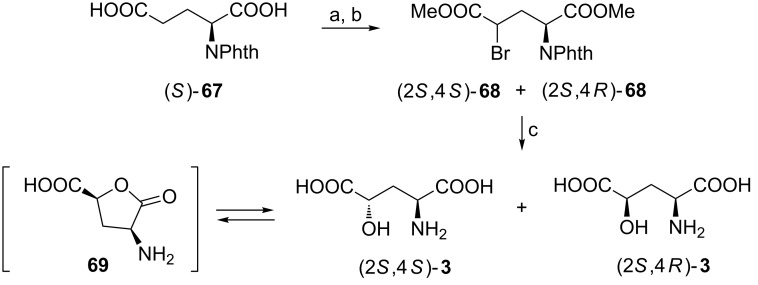
Synthesis of all stereoisomers of 4-hydroxyglutamic acid (**3**). Reagents and conditions: a) Br_2_, PBr_5_, *h*ν; b) MeOH, reflux; c) 6 M HCl, then pyridine, ethanol/water.

Bromination of *N*-phthaloyl-L-glutamic acid [(*S*)-**67**] followed by methanolysis gave a 2:1 mixture of *threo* and *erythro* diastereoisomers (2*S*,4*S*)-**68** and (2*S*,4*R*)-**68**, respectively (Scheme 17) [77–78]. A mixture of (2*S*,4*S*)-**3** and (2*S*,4*R*)-**3** obtained after hydrolysis was separated taking advantage of two phenomena: the preferential lactonization of (2*S*,4*S*)-**3** to produce **69** and much better solubility of (2*S*,4*R*)-**3** in water when compared with **69** and (2*S*,4*S*)-**3** [78]. Two other stereoisomers were synthesized in a similar way from (*R*)-**67**.

#### By a nitrone–acrylate cycloaddition

**Scheme 18:**
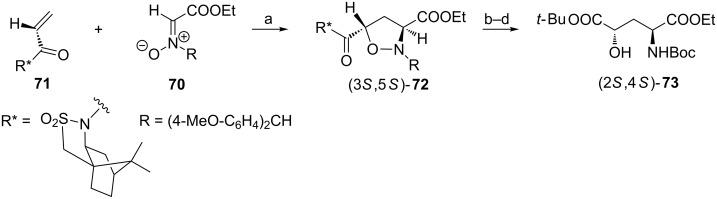
Synthesis of the orthogonally protected 4-hydroxyglutamic acid (2*S*,4*S*)-**73**. Reagents and conditions: a) toluene, 25 °C; b) LiOH, H_2_O_2_/THF; c) *O*-*tert*-butyl-*N*,*N*′-diisopropylisourea, CuCl; d) H_2_, 10% Pd/C, Boc_2_O, MeOH.

The isoxazolidine ring can be considered as another cyclic precursor to 4-hydroxyglutamic acids due to the easy cleavage of the N–O bond and high *trans* diastereoselectivities of 1,3-dipolar cycloadditions which allow to control stereochemistries at C3 and C5 [79–80]. To illustrate this concept the *E*/*Z* mixture of nitrone **70** was reacted with acrylamide **71** prepared from (2*S*)-bornane-10,2-sultam to afford mainly (20:1) the isoxazolidine (3*S*,5*S*)-**72** easily separable from minor cycloadducts. The *trans* stereochemistry of the isoxazolidine ring in **72** was the consequence of the *endo* and *exo* additions to the *Z*- and *E*-nitrones, respectively [80]. Further steps to the orthogonally protected (2*S*,4*S*)-**73** required selective hydrolysis of the chiral auxiliary, installation of the *tert*-butyl ester function and finally hydrogenolytic opening of the isoxazolidine ring with simultaneous protection of the amino group (Scheme 18).

#### By Diels–Alder reaction

**Scheme 19:**
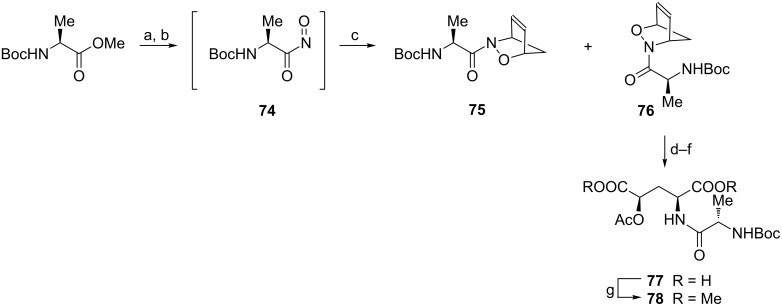
Synthesis of (2*S*,4*R*)-4-acetyloxyglutamic acid as a component of a dipeptide. Reagents and conditions: a) NH_2_OH, MeOH; b) Bu_4_NIO_4_, MeOH; c) cyclopentadiene, MeOH; d) Mo(CO)_6_, MeCN/H_2_O; e) Ac_2_O, pyridine/CH_2_Cl_2_; f) NaIO_4_, RuCl_3_, CCl_4_/MeCN/H_2_O; g) CH_2_N_2_, ether.

Acylnitroso derivative **74** prepared from methyl *N*-Boc-L-alaninate underwent Diels–Alder reaction with cyclopentadiene to produce equimolar amounts of easily separable cycloadducts **75** and **76** (Scheme 19) [81]. The bicyclic framework in the latter compound was first reduced and the hydroxy group was protected as acetate. Then the oxidative cleavage of the C=C bond gave diacid **77** (readily purified as dimethyl ester **78**) which is a dipeptide containing O-protected (2*S*,4*R*)-4-hydroxyglutamic acid **3** (Scheme 19). The cycloadduct **75** can be transformed in a similar manner into non-proteinogenic D-amino acids.

#### From 4-hydroxyproline

**Scheme 20:**
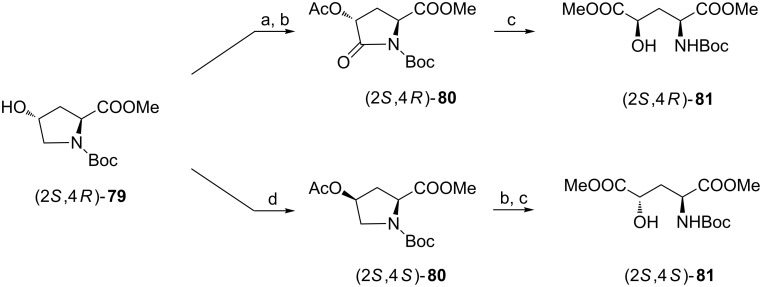
Synthesis of *N-*Boc-protected dimethyl esters of (2*S*,4*R*)- and (2*S*,4*S*)-**3** from (2*S*,4*R*)-4-hydroxyproline. Reagents and conditions: a) Ac_2_O, pyridine; b) RuO_2_, NaIO_4_, AcOEt/H_2_O; c) MeOH, K_2_CO_3_; d) AcOH, Ph_3_P, DEAD, THF.

4-Hydroxyproline could be used as a starting material in the chemical synthesis of 4-hydroxyglutamic acids when intermediary 4-hydroxypyroglutamic acids would have become available. This can be readily accomplished with ruthenium(IV) oxide. Application of this reagent to the acetate of methyl *N*-Boc-4-hydroxyprolinate [(2*S*,4*R*)-**79**] gave the protected pyroglutamate **80** which was transformed into dimethyl *N*-Boc-4-hydroxyglutamate [(2*S*,4*R*)-**81**] (Scheme 20) [82]. To synthesize (2*S*,4*S*)-**81** the inversion of configuration at C4 executed by Mitsunobu reaction preceded oxidation at C5 and the ring opening [82]. *O*-Benzyl ethers of (2*S*,4*R*)-**3** and (2*S*,4*S*)-**3** were prepared by the same methodology [50].

**Scheme 21:**
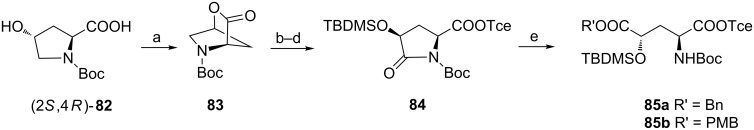
Synthesis of orthogonally protected (2*S*,4*S*)-**3** from (2*S*,4*R*)-4-hydroxyproline. Reagents and conditions: a) Ph_3_P, DEAD, THF; b) Cl_3_CCH_2_OH (TceOH), NaH, THF; c) *t*-BuMe_2_SiCl, imidazole, DMF; d) RuO_2_, NaIO_4_, CCl_4_/MeCN/H_2_O; e) BnOH or *p*-MeO-C_6_H_4_-CH_2_OH, NaH, THF.

Another approach to the orthogonally protected (2*S*,4*S*)-4-hydroxyglutamic acid **85** as an intermediate in the total synthesis of antibiotic nosiheptide [83–84] employs the *N-*Boc derivative of natural (2*S*,4*R*)-4-hydroxyproline **82** as a starting material (Scheme 21) [84–85]. The inversion of configuration at C4 was carried out by intramolecular lactonization to form **83** by implementation of the Mitsunobu reaction. After opening of the lactone ring with trichloroethanol and silylation of the hydroxy group oxidation at C5 was performed in the usual way to give a pyroglutamate **84**. Benzyl or *p*-methoxybenzyl esters **85a** or **85b** were next obtained after cleavage of **84** under basic conditions.

#### From pyroglutamic acid

**Scheme 22:**
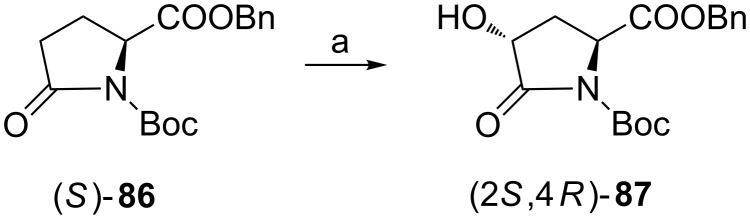
Synthesis of the protected (4*R*)-4-hydroxy-L-pyroglutamic acid (2*S*,4*R*)-**87** by electrophilic hydroxylation. Reagents and conditions: a) LiHMDS, THF, and then 3-phenyl-*N*-phenylsulfonyl oxaziridine.

In case of low availability of selected stereoisomers of 4-hydroxyprolines asymmetric syntheses of enantiomeric 4-hydroxypyroglutamates have been elaborated employing 1,3-dipolar cycloadditions of homochiral nitrones and acrylates [86–88] or a Diels–Alder reaction using acylnitroso compounds [89]. However, when compared with these multistep approaches hydroxylation of pyroglutamic acid derivatives seems to be the simplest option. Treatment of the lithium enolate of benzyl *N*-Boc-pyroglutamate (*S*)-**86** with Davis oxaziridine produced (2*S*,4*R*)-**87** (Scheme 22) [90–92]. HPLC investigation of the reaction mixture showed that (2*S*,4*S*)-**87** was not formed [90]. Stereospecific hydroxylation occurred on the opposite side to the benzyloxycarbonyl group, i.e., only *re*-face of the enolate was attacked for steric reasons. It is worth mentioning that hydroxylation of lithium enolates of pyroglutamate and glutamate results in the opposite stereochemical outcome at C4 (*R* vs *S*) and formation of a single diastereoisomer for the cyclic system and a 9:1 mixture for the linear one.

### 3,4-Dihydroxyglutamic acids

**Figure 5:**
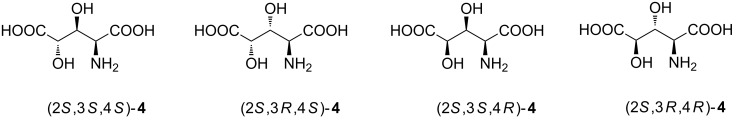
Enantiomers of 3,4-dihydroxy-L-glutamic acid (**4**).

Structures of enantiomers of 3,4-dihydroxy-L-glutamic acid (**4**) are depicted in Figure 5.

Several methodologies toward enantiomeric 3,4-dihydroxy-L-glutamic acid have been developed. In terms of carbon atom economy syntheses using 5-carbon synthons, e.g., pyroglutamic acid derivatives or pentoses, are the most valuable.

#### From pyroglutamic acid

**Scheme 23:**
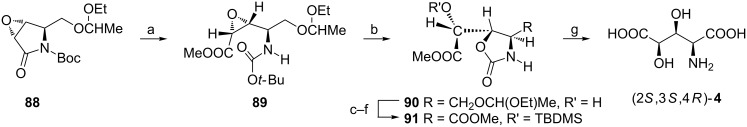
Synthesis of (2*S*,3*S*,4*R*)-**4** from the epoxypyrrolidinone **88**. Reagents and conditions: a) MeOH, THF, KCN (cat.); b) MeOH, SiO_2_; c) *t*-BuMe_2_SiCl, imidazole, DMF; d) 0.01 M HCl; e) Jones reagent; f) CH_2_N_2_, ether; g) 6 M HCl, reflux.

Cleavage of the 5-membered ring in the protected epoxide **88** obtained from (*S*)-pyroglutamic acid [93–95] gave the methyl ester **89** which, when adsorbed on silica gel, smoothly underwent stereospecific epoxide ring opening to give the oxazolidinone **90** (Scheme 23) [96]. Before installation of the second carboxylic group the secondary hydroxy group in compound **90** was transformed to the silyl ether while the hydroxymethyl fragment was subjected first to hydrolysis of the acetal, then to oxidation and esterification of the acid to provide **91**. After acidic hydrolysis (2*S*,3*S*,4*R*)-3,4-dihydroxyglutamic acid [(2*S*,3*S*,4*R*)-**4**] was obtained as the hydrochloride.

**Scheme 24:**
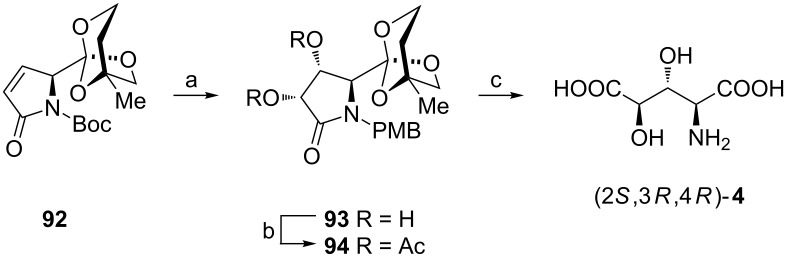
Synthesis of (2*S*,3*R*,4*R*)-**4** from the orthoester **92**. Reagents and conditions: a) OsO_4_, NMO, acetone/water, MeOH; b) Ac_2_O, pyridine; c) 1 M HCl, reflux, then Dowex 50W-X8.

To avoid racemization at Cα in sensitive amino acids the carboxy group was frequently masked as an orthoester. To illustrate this strategy dihydroxylation of the orthoester **92** (derived from L-pyroglutamic acid [97]) was performed to afford a single diastereoisomer **93** since the bulky orthoester residue allows the osmylation to occur from the opposite side (less hindered face). After purification of the diacetate **94** the recovery of acid (2*S*,3*R*,4*R*)-**4** was performed (Scheme 24) [98]. However, the hydrolysis was carried out under mild conditions to prevent decomposition of this stereoisomer including racemization at Cα.

#### From pentose via 2,3-aziridino-γ-lactone

**Scheme 25:**
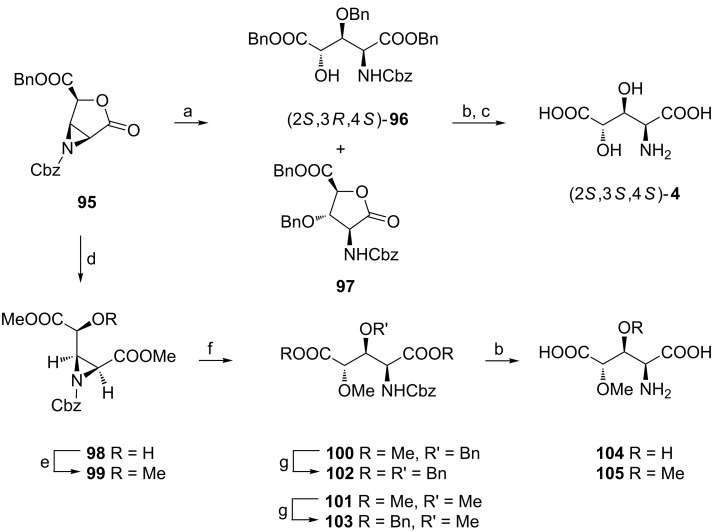
Synthesis of (2*S*,3*S*,4*S*)-**4** from the aziridinolactone **95**. Reagents and conditions: a) BnOH, BF_3_**·**OEt_2_, CHCl_3_; b) H_2_, 10% Pd/C, MeOH, then MeOH, H_2_O; c) HO^–^ resin AG1-X4; d) MeOH, BF_3_**·**OEt_2_; e) MeI, Ag_2_O, CaSO_4_, THF; f) BnOH or MeOH, BF_3_**·**OEt_2_; g) Ti(OBn)_4_, BnOH, toluene.

In the so called “2,3-aziridino-γ-lactone methodology” [18,99–100] ribose (or lyxose) is used as a starting material [101–102] which is transformed into the lactone **95** in several steps [99]. Boron trifluoride etherate-catalyzed reaction of **95** with benzyl alcohol induces first opening of the 5-membered ring to form a benzyl ester and later the cleavage of the 3-membered ring to give a vicinal *N-*Cbz aminoalcohol with inversion of configuration. However, the reaction mixture (1:1) consists of the protected 3,4-dihydroxy-L-glutamic acid **96** and the respective γ-lactone **97** formed from **96** in the presence of acids. Benzyl alcohol was selected to refrain from decomposition of the final amino acids during the acid hydrolysis of, e.g., methyl esters [99] since for a mixture of **96** and **97** hydrogenolysis cleanly liberates (2*S*,3*S*,4*S*)-**4** (Scheme 25) [100].

This methodology opens the way to (3*S*,4*S*)-3-hydroxy-4-methoxy- (**104**) and 3,4-dimethoxy-L-glutamic acid (**105**) since selective opening of the lactone ring in **95** can be accomplished by boron trifluoride etherate-catalyzed methanolysis at low temperatures to give dimethyl ester **98** (Scheme 25). Silver oxide-promoted methylation introduces a MeO-C4 unit. Regioselective aziridine ring opening in **99** was then carried out in the known way with benzyl alcohol or methanol to produce substituted dimethyl L-glutamates **100** and **101**. To ensure clean deprotection in the final step transesterification of methyl to benzyl esters was successfully performed in the presence of titanium(IV) benzyloxide to afford dibenzyl esters **102** and **103**, respectively. Their hydrogenolysis cleanly produced (2*S*,3*S*,4*S*)-**104** and (2*S*,3*S*,4*S*)-**105** [103].

#### From tartaric acids

**Scheme 26:**
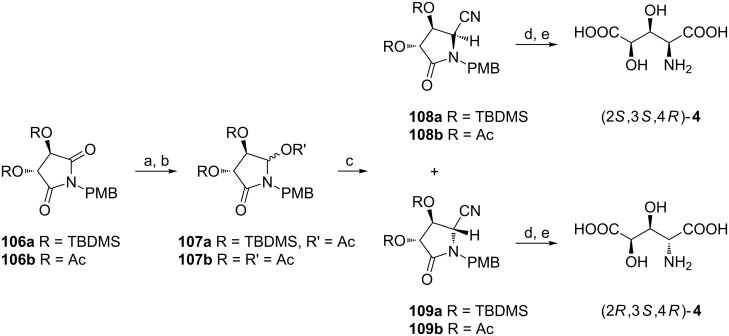
Synthesis of (2*S*,3*S*,4*R*)-**4** and (2*R*,3*S*,4*R*)-**4** from cyclic imides **106**. Reagents and conditions: a) NaBH_4_, MeOH; b) Ac_2_O, pyridine; c) Me_3_SiCN or Bu_3_SnCN, BF_3_**·**OEt_2_, toluene or CH_2_Cl_2_; d) Ce(NH_4_)_2_(NO_3_)_6_, MeCN/H_2_O; e) 6 M HCl, reflux, then Dowex 50W-X8.

Four-carbon chirons derived from D- or L-tartaric acids can be used as starting materials in syntheses of enantiomers of 3,4-dihydroxyglutamic acids since they contain a vicinal diol fragment of the known stereochemistry. To demonstrate this strategy cyclic imides **106a** (R = TBDMS) and **106b** (R = Ac) readily prepared from L-tartaric acid [104–105] were reduced and the respective hydroxylactams were acetylated to produce acetoxylactams **107a** and **107b**, necessary intermediates in the next step (Scheme 26) [106–107]. The introduction of the cyano group was accomplished by boron trifluoride-catalyzed reaction with trimethylsilyl or tributyltin cyanides and the stereochemical outcome of these reactions strongly depends on the protecting group. Diastereoisomeric excesses of 60–80% were observed in the cyanation of *tert*-butyldimethylsilyl ether **107a** and **108a** was the major product, while for the acetate **107b** the selectivity was lower (de 54–64%) with **109b** predominating. Nevertheless, efficient separation of the diastereoisomers was achieved for **108a** and **109a** only and they were deprotected to give enantiomerically pure (2*S*,3*S*,4*R*)-**4** and (2*R*,3*S*,4*R*)-**4**, respectively. Application of D-tartaric acid as a starting material provided (2*S*,3*R*,4*S*)-**4** and (2*R*,3*R*,4*S*)-**4**.

**Scheme 27:**
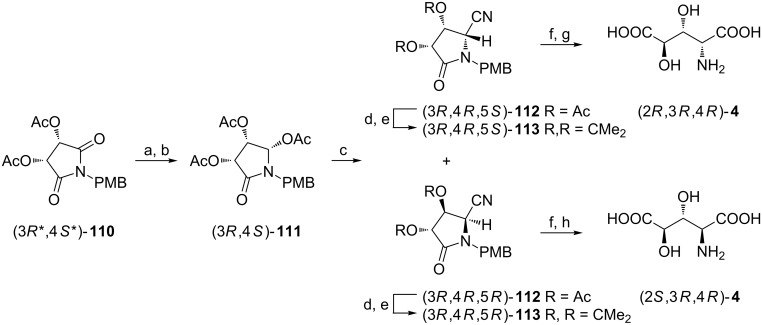
Synthesis of (2*R*,3*R*,4*R*)-**4** and (2*S*,3*R*,4*R*)-**4** from the cyclic *meso*-imide **110**. Reagents and conditions: a) (*R*)-BINAL-H (EtOH), THF; b) Ac_2_O, pyridine; c) Me_3_SiCN, BF_3_**·**OEt_2_, toluene; d) AcCl, EtOH; e) Me_2_C(OMe)_2_, acetone, PTSA; f) Ce(NH_4_)_2_(NO_3_)_6_, MeCN/H_2_O; g) 6 M HCl, reflux, then Dowex 50W-X8; h) 1 M HCl, reflux, then Dowex 50W-X8.

To secure the (3*R*,4*R*) and (3*S*,4*S*) configurations in 3,4-hydroxyglutamic acids enantioselective reduction of the carbonyl group of the cyclic imide (3R*,4S*)-**110** prepared from *meso*-tartaric acid [108] needs to be elaborated (Scheme 27) [109]. Low temperature reduction of this imide with a reagent obtained from (*R*)-binaphthol [(*R*)-BINAL-H] followed by acetylation furnished the triacetate (3*R*,4*S*)-**111** as a single diastereoisomer after chromatographic purification (Scheme 27). However, its cyanation as described earlier gave a 38:62 mixture of diacetates (3*R*,4*R*,5*S*)-**112** and (3*R*,4*R*,5*R*)-**112** which were separated as isopropylidene derivatives (3*R*,4*R*,5*S*)-**113** and (3*R*,4*R*,5*R*)-**113**. After deprotection they were converted into (2*R*,3*R*,4*R*)-**4** and (2*S*,3*R*,4*R*)-**4**, respectively, although the final hydrolytic step in the synthesis of (2*S*,3*R*,4*R*)-**4** had to be carried out carefully since its instability to concentrated acid. Enantiomers (2*S*,3*S*,4*S*)-**4** and (2*R*,3*S*,4*S*)-**4** were obtained in a similar way using (*S*)-BINAL-H as a reducing agent, thus completing syntheses of all eight stereoisomers of 3,4-dihydroxyglutamic acid.

#### From D-serine

**Scheme 28:**
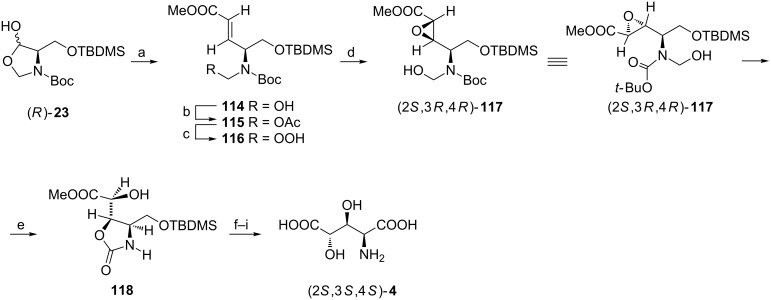
Synthesis of (2*S*,3*S*,4*S*)-**4** from the protected serinal (*R*)-**23**. Reagents and conditions: a) Ph_3_P=CHCOOMe, benzene; b) Ac_2_O, NEt_3_, DMAP, CH_2_Cl_2_; c) 30% H_2_O_2_, PTSA, MgSO_4_, DME; d) K_2_CO_3_, MeOH; e) MeOH, 45 °C; f) Piv_2_O, NEt_3_, DMAP, CH_2_Cl_2_; g) PTSA, MeOH; h) CrO_3_, H_5_IO_6_, MeCN; i) 6 M HCl, reflux.

An interesting strategy to (2*S*,3*S*,4*S*)-**4** (Scheme 28) [110] employs a protected serinal (*R*)-**23** [54]. Wittig olefination extended the alkyl chain by two carbon atoms and simultaneously installed the C=C bond which was subjected to the intramolecular epoxidation to give a >20:1 mixture of aminoepoxides with the isomer (2*S*,3*R*,4*R*)-**117** dominating. Without isolation this compound underwent another intramolecular cyclization in the 5-*exo* mode to form the oxazolidinone **118**. To complete the synthesis of (2*S*,3*S*,4*S*)-**4** the secondary hydroxy group was protected as a pivalate, the hydroxymethyl fragment was oxidized after hydrolysis of the silyl ether and finally all protecting groups were removed by concentrated acid.

**Scheme 29:**
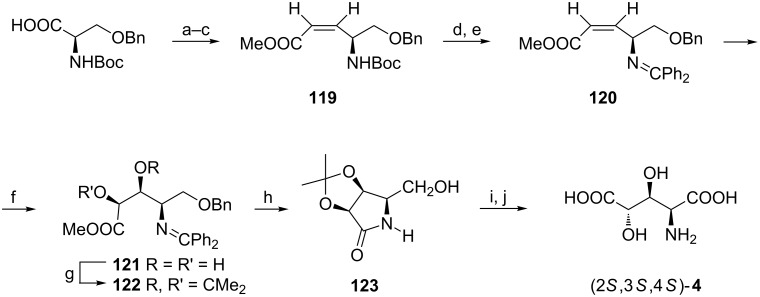
Synthesis of (2*S*,3*S*,4*S*)-**4** from *O*-benzyl-*N*-Boc-D-serine. Reagents and conditions: a) ClCOOiBu, TEA, DME, then NaBH_4_, MeOH; b) Dess–Martin periodinate, CH_2_Cl_2_; c) (CF_3_CH_2_O)_2_P(O)CH_2_COOMe, KHMDS, 18-crown, THF; d) AcCl, MeOH; e) Ph_2_C=NH, CH_2_Cl_2_; f) OsO_4_, NMO, THF/H_2_O; g) Me_2_C(OMe)_2_, PPTS, benzene; h) H_2_, 20% Pd(OH)_2_, MeOH; i) RuCl_3_, NaIO_4_, CCl_4_/MeCN/H_2_O; j) 6 M HCl, 80 °C.

A very efficient synthesis of (2*S*,3*S*,4*S*)-**4** starts from another serine-derived chiron, namely *O*-benzyl-*N*-Boc-D-serine [111], which was readily transformed to the *Z*-olefin **120** containing a benzophenone imine residue as a nitrogen protecting group (Scheme 29). Dihydroxylation of the C=C bond gave a 10:1 mixture with (2*S*,3*S*,4*R*)-**121** as a major product which was transformed into the isopropylidene derivative (2*S*,3*S*,4*R*)-**122** to facilitate purification. Hydrogenolysis allowed to remove the N- and O-protecting groups and was followed by the spontaneous cyclization to a pyrrolidine-2-one (3*S*,4*S*,5*R*)-**123** [111]. Oxidation of the hydroxymethyl group and acid hydrolysis gave (2*S*,3*S*,4*S*)-**4** [112].

#### By enantioselective conjugate addition and asymmetric dihydroxylation

**Scheme 30:**
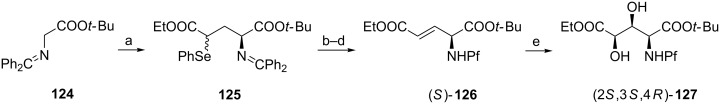
Synthesis of (2*S*,3*S*,4*R*)-**127** by enantioselective conjugate addition and asymmetric dihydroxylation. Reagents and conditions: a) ethyl 1-phenylselenylacrylate, chiral PTC, 50% KOH, CH_2_Cl_2_; b) 1 M HCl, THF; c) 9-bromo-9-phenylfluorene, K_2_PO_4_, PbNO_2_, MeCN; d) NaIO_4_, NaHCO_3_, MeOH/H_2_O; e) K_2_Os_2_(OH)_4_, hydroquinine 4-chlorobenzoate (HQN-CLB), K_2_CO_3_, K_3_Fe(CN)_6_, *t*-BuOH/H_2_O.

An orthogonally protected 3,4-dihydroxy-L-glutamic acid was envisioned as an intermediate in the projected synthesis of (+)-polyoxamic acid (Scheme 30) [113]. To this end the anion generated from benzophenone imine of *tert*-butyl glycinate **124** acted as a Michael donor in the presence of homochiral catalyst to give a ca. 1:1 mixture of diastereoisomeric iminoselenides **125** with an ee up to 96% (Scheme 30). Next, a 9-phenylfluorenyl protecting group was installed to prevent racemization and oxidation allowed to introduce the C=C bond leading to 3,4-didehydroglutamate (*S*)-**126**. Asymmetric dihydroxylation of (*S*)-**126** (ee 96%) gave (2*S*,3*S*,4*R*)-**127** (de 94%).

#### Synthetic applications of enantiomeric hydroxy-L-glutamic acids

**Figure 6:**
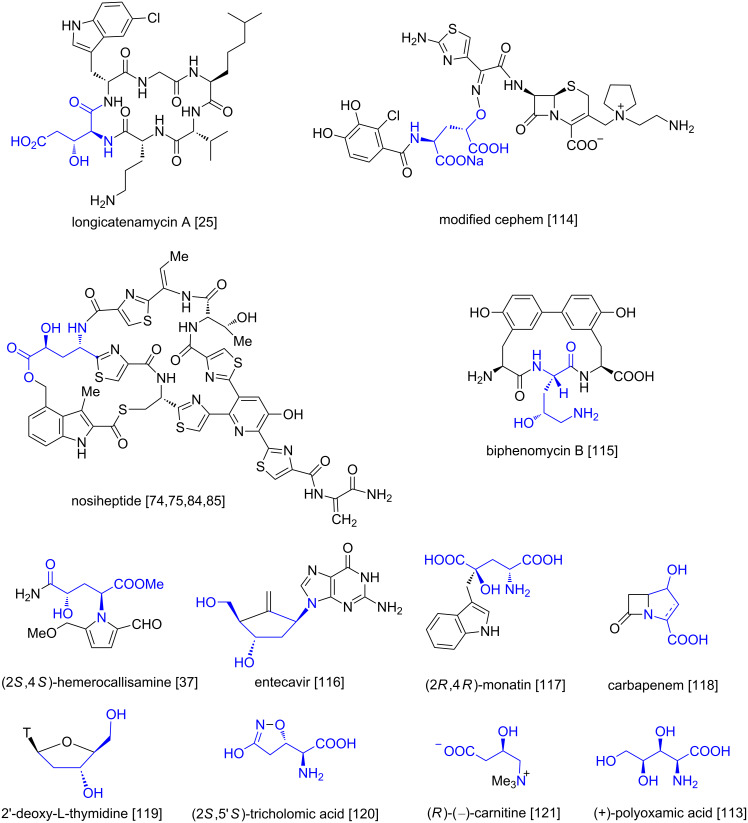
Structures of selected compounds containing hydroxyglutamic motives (in blue).

Besides numerous applications of hydroxyglutamic acids in studies on glutamate receptors they have also been used as starting materials in syntheses of other compounds including complex natural products (Figure 6).

Thus, (2*S*,3*R*)-5-(benzyloxy)-2-[(*tert*-butoxycarbonyl)amino]-3-hydroxy-5-oxopentanoic acid served as a precursor in the total synthesis of longicatenamycin A [25]. Syntheses of several modified cephems started from dimethyl (2*S*,4*R*)-*N*-Boc-4-hydroxyglutamate (**81**) [114]. Protected 4-hydroxyglutamic acids (2*S*,4*S*)-**66** [75–76] and (2*S*,4*S*)-**85a** [84–85] after installation of the thiazole ring at the C1 terminus were incorporated into the thiopeptide antibiotic nosiheptide. The total synthesis of biphenomycin B relied on installation of a five-membered chain derived from a protected *tert*-butyl (2*S*,4*R*)-4-hydroxypyroglutamate [115]. While the protected methyl (2*S*,4*S*)-4-hydroxypyroglutamate formed a basis for the construction of the alkaloid hemerocallisamine skeleton [37], its *tert*-butyl counterpart was used as a starting material in a multistep synthesis of a functionalized *exo*-methylenecyclopentane skeleton as an entecavir intermediate [116]. On the other hand, the stereospecific alkylation of methyl (2*R*,4*R*)-4-hydroxypyroglutamate was employed in the important approach to (2*R*,4*R*)-monatin [117]. Carbapenems can be generated from the intermediary enantiomeric 3-hydroxy- or 4 hydroxy-L-glutamic acids in cell-free environments [118]. Diazotization of (2*R*,3*R*)-**2** provided 2-deoxy-L-1,4-ribonolactone which was later transformed into 2'-deoxy-L-thymidine and other nucleosides [119]. Syntheses of all four enantiomers of tricholomic acid of interest as a flycidal compound as well as in receptor studies [120] were accomplished starting from enantiomeric 3-hydroxyglutamic acid, e.g., (2*S*,3*R*)-3-hydroxyglutamic acid was converted in a few steps into (2*S*,5'*S*)-tricholomic acid [47]. The absolute configuration of (*R*)-(−)-carnitine was established by enzymatic decarboxylation of (2*S*,3*R*)-3-hydroxyglutamic acid followed by exhaustive methylation [121]. Fluoroglutamic acids are of special interest for PET imaging [122] and among other methods they are available from 3- or 4-hydroxyglutamic acids by direct hydroxy to fluoride displacement [91,123–124]. Deuterium- [72] and tritium-labelled [125] glutamic acids have also been prepared from 4-hydroxyglutamic acids via 4-mesyloxy derivatives. And finally reduction of the ethoxycarbonyl group in (2*S*,3*S*,4*R*)-**127** followed by acidic hydrolysis gave (+)-polyoxamic acid [113].

## Conclusion

The synthesis of nonracemic hydroxyglutamic acids is an active area of research and it was stimulated by studies on glutamate receptors to modulate the biological activity of L-glutamic acid from one side and applications as starting materials in total syntheses of complex natural products from the other. In general, the syntheses started from other amino acids and were designed to preserve the stereochemical integrity at Cα while inducing chirality at Cβ- or Cγ-OH centers. Thus, both enantiomers of serine or their synthetic equivalents, glutamic acid, 4-hydroxyproline and pyroglutamic acid were most frequently employed. Alternatively, α-hydroxy acids (malic, tartaric) offered the opportunity to induce chirality at Cα–N while the stereochemistry at C–OH was retained. Monosaccharides (glucose, ribose) also appeared attractive providing two or three predefined stereogenic centers. In more sophisticated approaches application of chiral auxiliaries allowed to generate vicinal or 1,3-aminoalcohol units of the required stereochemistries.

Currently available synthetic methodologies towards hydroxyglutamic acids significantly differ in terms of carbon atom economy and preparative simplicity although carbon-wasteless approaches do exist. For future use as starting materials in total syntheses of complex natural products synthetic methodology to orthogonally protected hydroxyglutamic acids were also discussed which allow, for example, to differentiate between α and ω carboxy groups.

Although syntheses of particular enantiomers of hydroxyglutamic acids look to be optimal, e.g., (2*S*,4*S*)-**3** or (2*S*,4*R*)-**3** via hydroxylation of the protected glutamic or pyroglutamic acids, synthetic methodologies to the other enantiomers may require improvements or even designing new ways especially when larger quantities are needed and we hope this review will stimulate further research in this area.
